# Microfluidic train station: highly robust and multiplexable sorting of droplets on electric rails

**DOI:** 10.1039/c6lc01544a

**Published:** 2017-03-14

**Authors:** Daniel Frenzel, Christoph A. Merten

**Affiliations:** European Molecular Biology Laboratory (EMBL), Genome Biology Unit, Heidelberg, Germany

## Abstract

Fluorescence-activated droplet sorting (FADS) has become a widely used technique for high-throughput screening applications. However, existing methods are very sensitive to fluctuating flow rates at the sorting junction, which can be caused by the pulsing effects of mechanical pumps, droplet aggregates or the accumulation of precipitates during lengthy biological screening applications. Furthermore, existing sorting devices allow only 2-way sorting. We present here a dielectrophoretic sorting system in which the droplets are sorted along multiple electrode pairs that run parallel to the channels. This enables highly reliable sorting (no errors were detected for more than 2000 sorting events) even when inverting the relative flow rates at a 2-way sorting junction from 80 : 20 to 20 : 80. Furthermore, our toolbox is scalable: we demonstrate on the example of a triple-colour sorting experiment with a total of four decoupled electrodes that multi-way sorting is feasible.

## Introduction

Droplet-based microfluidics holds great promise for high-throughput screening applications.^1^,^2^ One of its key advantages is the fact that entire assay vessels containing soluble molecules can be sorted according to a desired readout (*e.g.* the fluorescence signal), rather than just cells or other solid particles as in a FACS sorter.^3^ This has been exploited in manifold applications such as antibody or drug screening applications, genetic assays and directed evolution.^4^–^8^ Microfluidic droplet sorting is usually based on dielectrophoresis or acoustophoresis and enables the manipulation of samples at up to kilohertz frequencies.^9^,^10^ The typical sorting setup consists of a sorting junction where, in the absence of any electric or acoustic field, all droplets are sent to a designated waste output channel. This can be achieved by adjusting the hydrodynamic resistance of the collection channel (using a higher resistance in comparison with that of the waste channel), or by aspirating an asymmetric fraction of the total flow from one of the two channels (*e.g.* using a syringe pump in the refill mode to aspirate 70% of the total flow from the waste outlet). To collect a specific droplet, an electric or acoustic pulse is applied, thus pulling or pushing the desired droplet into the hydrodynamically less favoured collection channel.

So far, this basic sorting principle has been used for more than ten years. Nonetheless, it has two inevitable limitations: first of all, the approach is very sensitive to minor variations in the fluid flow and the resistance of individual channel sections. The frequently observed pulsing of syringe pumps (having threads which on the microscale cannot be rotated at a 100% constant speed) or the formation of droplet aggregates in widening channels can push non-selected droplets into the collection channel. Similarly, deposits in the channels (dust particles or protein precipitates) can change the fluidic resistance, so that undesired droplets are collected. In consequence, it is difficult to run microfluidic droplet sorting experiments stably over many hours. However, this is required for many biological screening applications in which hundreds of thousands or even millions of droplets have to be sorted based on the endpoint signal of biochemical or cell-based fluorescence assays. Using current approaches which are sensitive to fluctuations in the relative flow rates at the sorting junction this inevitably results in the selection of false positives. For example, on screening more than 300 000 hybridoma cells over a period of ∼4 h, we previously reported a false positive rate of more than 5%.^4^ Similarly other groups have demonstrated 3–4 hour sorting experiments focussing on metabolic^11^ or genetic^6^ assays, which could strongly profit from a systematic reduction of the false positive rate.

Another limitation of existing sorting devices is the fact that they hardly allow sorting into more than two channels. This is a significant disadvantage, since multi-way sorting has tremendous potential for screening applications. For example, it could be used to sort samples according to the strength of a phenotype (*e.g.* very strong = collection channel 1, strong = collection channel 2, intermediate = collection channel 3, *etc.*) rather than just in a digital on/off fashion. Only in this way can truly quantitative and mechanistic data be obtained from downstream genetic or biochemical analyses of the sorted samples. Alternatively multichannel sorting can be used in multiplexed assays (*e.g.* sorting droplets according to more than one phenotype). However, existing sorting devices do not allow these operations to be performed on microfluidic droplets.

We have overcome these limitations using dielectrophoretic sorting devices that have an electrode pair not only on the side of the collection channel, but also on the side of the waste channel. Furthermore, in our approach, exactly one electrode pair is activated at any point in time: whenever a droplet is showing a desired readout signal, the electrode pair next to the collection channel is switched on, while for all other times, only the electrode pair next to the waste channel is switched on. This enables reliable sorting without the need to adjust the relative flow rates at the sorting junction (and also without being sensitive to flow rate fluctuations) and furthermore allows multi-way sorting by placing electrode pairs next to each collection channel. This enables an unprecedented level of control by moving droplets on “electric rails”.

## Results

**Fig. 1:**
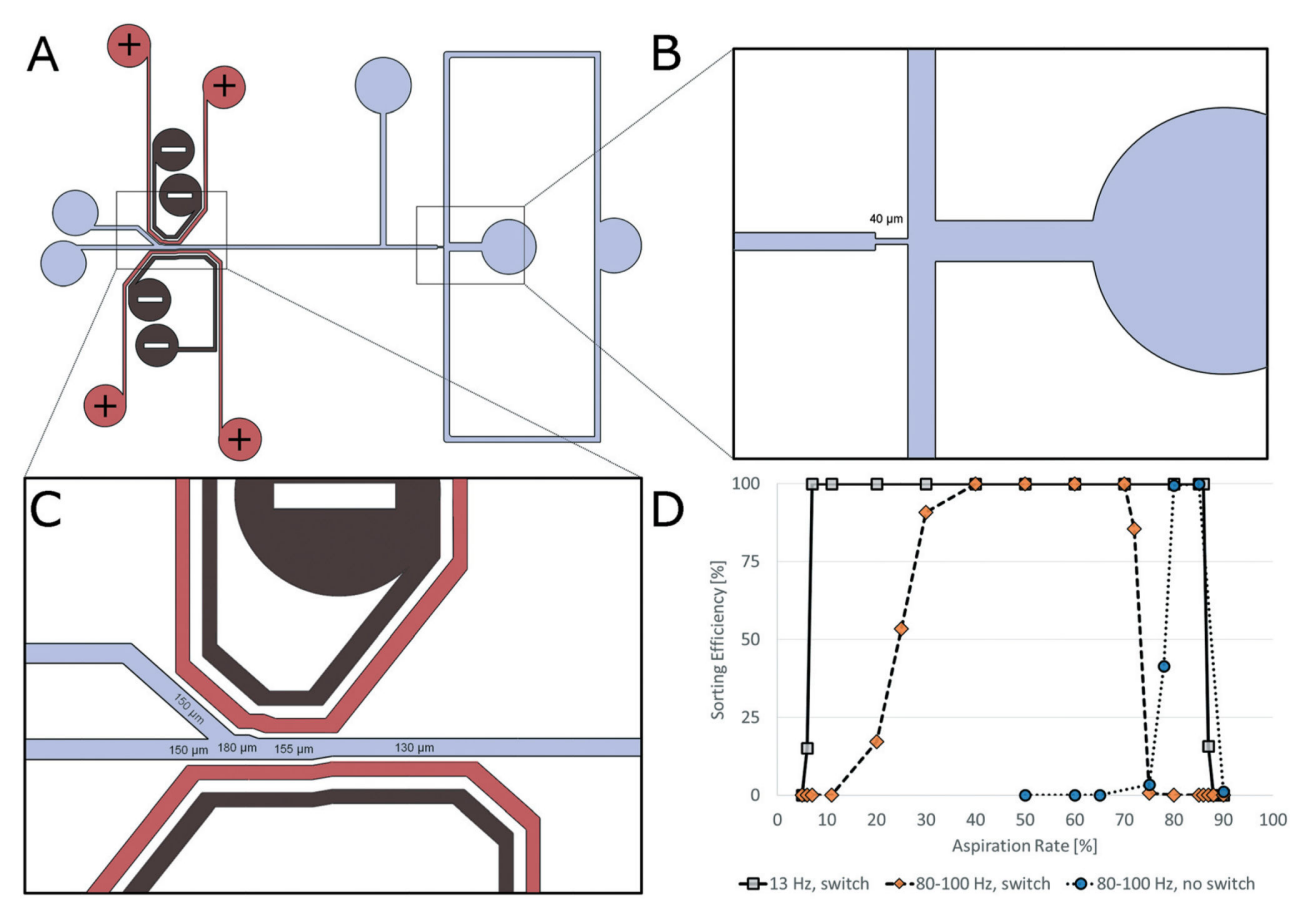
2-Way sorting on electric rails. A) Design of the 2-way sorting chip. The chip contains two pairs of parallel electrodes. Ground electrodes are indicated by a “−” sign, and power electrodes are indicated by a “+” sign. B) Zoomed-in image of the droplet maker. The nozzle has a diameter of 40 μm. C) Zoomed-in image of the sorting junction with two electrode pairs on either side of the channel. The electrodes follow the shape of the channel in a constant distance of 50 μm. D) Sorting efficiency at different relative flow rates in the waste and collection channels. Indicated percentages (grey: 5–88% for 13 Hz sorting; orange: 25–65% for 90 Hz sorting and blue: 60–80% for 90 Hz sorting with just one electrode pair) of the total input flow rate were aspirated from the straight “waste” channel.

The design of our 2-way sorting device is shown in Fig. 1A–C. We are using electrodes running parallel to the channel walls. For sorting, the waste electrode pair is switched on unless a droplet with the desired properties passes by. In this case, the waste electrode pair is switched off and the collection electrode pair is switched on for a time interval sufficiently long to guide the desired droplet into the collection channel (typically 5–10 ms). Immediately afterwards, all electrodes are switched back to the initial configuration. The reliability of this sorting setup is further improved by the fact that both electrode pairs are passing the sorting divider. Hence the trajectory of the droplets can be controlled not only at the sorting junction, but also at all times until they have irreversibly entered the desired channel.

To demonstrate the feasibility of this approach, we sorted 100 μm droplets containing 100 μM FITC. To analyse the sorting efficiency, we used an algorithm triggering the electrodes only for every second, third or fifth droplet. This allowed accurate monitoring of the correct sorting of individual droplets by video analysis (Table 1), revealing 100% efficiency. This high level of robustness was further confirmed by sorting every second droplet over a period of five hours, during which videos were taken every hour. On analyzing a total of 4659 sorting events, not a single mistake was observed (Table S1†). After optimizing all flow rates, we further achieved a maximum throughput of ∼240 Hz (Movie S1†), which is approximately 5-fold higher than what we achieved previously for droplets of the same size, using a conventional sorting setup.^4^ More importantly, we could show that the sorting efficiency is largely independent of the relative flow rates downstream of the sorting junction. By connecting a syringe aspirating fluid from the waste outlet (while leaving the collection outlet unaffected), we aspirated different fractions of the overall flow and monitored the sorting efficiency (using the algorithm for sorting every second droplet) by high-speed imaging (Movies S2–S7†). Without changing any other sorting parameters (*e.g.* pulse amplitude and/or duration), the sorting efficiency remained completely unaffected at ∼100% (see details in Table 2 and Fig. 1D), while aspirating from 7% to 86% (at 13 Hz) and 40% to 70% (at ∼90 Hz) in terms of the relative flow rates at the waste channel. In contrast, sorting with just one electrode pair (mimicking a conventional setup) worked well when constantly aspirating 80% of the relative flow from the waste outlet, but failed when changing the relative flow rates by more than 5% (Table 2, Fig. 1D and Movies S8–S10†). Similarly, our setup showed much higher robustness when perturbing the system in a real world context; while performing sorting experiments with one and two electrode pairs (at flow rates which usually allow 100% efficiency in both systems; 80 : 20 for one and 50 : 50 for two electrode pairs, see Fig. 1D), we gently flicked the tubing connected to the collection outlet. This procedure mimics a perturbation that occurs frequently in long-term biological screens, such as moving the tubing between different collection tubes (*e.g.* to avoid a single mistake that can ruin an entire screen of valuable samples or when changing sorting gates). For each experiment, the tubing was gently flicked six times within six seconds and the entire process was recorded using high-speed imaging (ESI† Movies S11 and S12). Furthermore, each experiment was repeated three times to obtain high statistical confidence (Table 3). While for the single electrode pair setup the perturbation decreased the sorting efficiency on average to 92.2%, the two electrode setup allowed 99.4% efficiency (analyzing more than 2600 sorting events in both cases), even under such difficult conditions. These values correspond to a 13-fold reduced number of false positives.

To complete the characterization of the 2-way sorting device, we finally had a look at the effect of the constant DEP force on the droplet velocity (Table S2 and Movie S13†) and the ability of our device to sort droplets of different sizes without changing the geometry (Fig. S6 and Movies S7, S12 and S14†). In brief, the constant electric field decreased the droplet velocity very little (by a maximum of 9.4%, even when applying 1688 V rather than 1500 V as done for the sorts) and this is valid over a wide range of different total flow rates. Furthermore, the device enabled reliable sorting of droplets with sizes ranging from approximately 60–120 μm in diameter (corresponding to an 8-fold difference in volume), without changing the geometry. Larger or smaller droplets can probably be sorted as well, but could not be generated using a droplet maker of constant size.

**Fig. 2:**
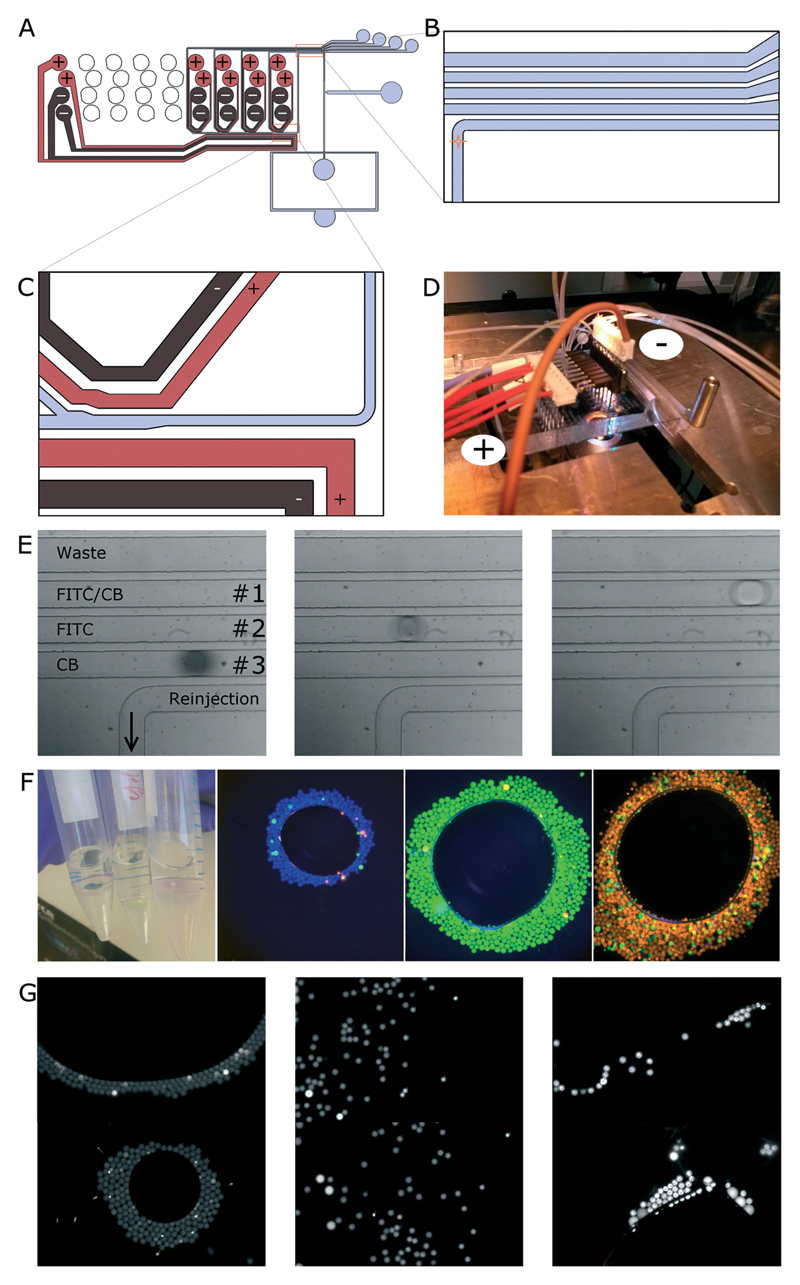
4-Way sorting on electric rails. A) Design of the 4-way sorting chip. B) Zoomed-in image of the detection area. Each droplet is detected when passing the focus of three aligned lasers (indicated by an orange star). C) Zoomed-in image of the first sorting junction. The droplets move along the powered waste electrode and slow down in the region with increasing channel diameter. If the sorting criterion for a particular collection channel is fulfilled, the respective sorting electrode pair is powered instead of the waste electrode pair and the droplets are hence pulled into the respective collection channel. Otherwise the waste electrode pair remains powered and the droplets move on to the next sorting junction, where the process is repeated until the droplets reach the desired collection or waste channel. D) Multi-way sorting setup. The electrodes are connected *via* standard 2.54 mm pin headers. The horizontal pin headers connected to the red wires (+) correspond to the signal lines. The vertical pin headers connected to the brown wire (−) correspond to ground. E) Multi-way sorting of droplets hosting (a combination of) two different fluorophores. An emulsion containing droplets hosting 187.5 μM cascade blue (CB), 250 μM fluorescein (FITC), and a combination of both (187.5 μM FITC, 125 μM CB) was reinjected into the chip for sorting. To distinguish the droplet fractions during imaging, naphthol blue black was added to the droplets containing CB (dark grey) or FITC (bright grey). The droplets were then sorted into the respective collection channels as indicated on the left image. F) Multi-way sorting of droplets hosting three different fluorophores. An emulsion composed of droplets containing 187.5 μM CB (blue), 20 μM Alexa Fluor 488 (green) and 100 μM Alexa Fluor 594 (red) was generated and reinjected into the sorting device. After 13 h of continuous operation, aliquots of the sorted fractions were imaged in bulk (left) or by fluorescence microscopy (right). G) Multi-way sorting of droplets hosting three different concentrations of Alexa Fluor 488 (12.5 μM, 25 μM and 50 μM). Subsequent to droplet generation, the mixed emulsion was reinjected into the sorting device. After 8 h of continuous operation, aliquots of the three sorted fractions (increasing concentrations from left to right) were imaged by fluorescence microscopy. Two fields of view were merged for each concentration.

Next, we focused on multi-way sorting. This was implemented by designing a sorting module (Fig. 2A–C) in which four channels sequentially branch off from the waste channel. Each of these collection channels has its own electrode pair running parallel to the channel wall (Fig. 2C). In order to simplify the wiring, we spaced out the connection points for the electrodes to match low cost 2.54 mm pin connectors (Fig. 2D).

In a first experiment, we focussed on high-speed analysis of the sorting process, using red light to illuminate the chip (so that interference with the photomultiplier tubes of the green and blue channels can be ruled out). For this purpose, we produced droplets containing cascade blue (CB), fluorescein isothiocyanate (FITC), and a combination of both (using the droplet maker shown in ESI† Fig. S1; see the Materials and methods section for details on the dye concentrations) and performed dual-colour sorting using a multi-channel optical setup (Fig. S2†). To be able to monitor the sorting efficiency, the droplets were additionally stained with different concentrations of the chromogenic dye naphthol blue black, allowing the different droplet populations to be distinguished by the eye and even outside the laser spot (Fig. 2E and Movie S15†). After determining the optimal pulse durations and – delays (the further downstream a particular collection channel is located, the longer the pulse delay; Tables 2 and 3), we sorted a reinjected emulsion containing the different droplet populations for a total of 2 hours and measured a sorting efficiency of >98% (averaged over all sorting channels in two independent experiments) at a sorting rate of ∼2 Hz.

We then repeated the experiment and determined the long-term sorting efficiency based on subsequent fluorescence microscopy analysis of the sorted fractions. This readout is not dependent on high-speed movies, for which reason we could dim the red light used for illumination, thus allowing the detection of a third red droplet species. We hence generated droplets containing cascade blue (CB), Alexa 488, or Alexa 594, reinjected the mixed emulsion into the sorting device and sorted unattended over 13 hours. During this time, an overall throughput of 3 Hz at ∼90% efficiency (average value based on microscopic analysis of all three sorted fractions; Fig. 2F and Table 3) was achieved. This long-term efficiency clearly demonstrates the suitability of our system, not only for short proof-of-principle experiments, but also for real screening campaigns.

As a final application, we further addressed multi-way sorting according to the signal intensity. Instead of using different fluorophores, we encapsulated different concentrations of Alexa 488 (12.5 μM, 25 μM and 50 μM) and sorted the droplets according to their fluorescence intensity in the green channel. After sorting for a total of eight hours, aliquots from the different collection tubes were taken and analyzed by fluorescence microscopy. Similar to the previous experiments, an overall throughput of 3 Hz at ∼90% efficiency (average value based on microscopic analysis of all three sorted fractions; Fig. 2G and Table 5) was achieved. This clearly demonstrates the possibility of sorting samples not just for different phenotypes, but as well as for the quantitative strength of a particular phenotype.

## Discussion

We present here a dielectrophoretic sorting setup that has two main advantages over previous approaches: by using multiple electrode pairs that run parallel to the channels, we achieve ∼100% reliable 2-way sorting, largely independent of the relative flow rates in the channels downstream of the sorting junction. Hence sorting can be performed efficiently and unattended overnight, and factors causing fluctuations in the flow rates become negligible. This is particularly relevant when using mechanical syringe pumps (*e.g.* for reinjecting an emulsion, spacing with oil or simply for adjusting the relative flow rates at the sorting divider^4^), which always tend to show a pulsing behaviour. Furthermore, our setup can be used to overcome changes in channel resistances, either caused by droplet aggregates in the collection channel or by precipitates as frequently observed for long-term sorting of protein-rich droplets (*e.g.* samples containing cell culture media). Taken together, we expect that our approach will significantly reduce the number of false positives in biological screens.

In addition, we present a toolkit which enables the experimenter to setup a scalable platform for highly multiplexed sorting applications at a slightly lower efficiency of 94% on average (based on almost 2000 analyzed sorting events shown in Tables 4 and 5). By using a total of four decoupled electrode pairs (three for specific sorting channels and one for the waste), we were able to sort three different droplet types, either containing unique dyes (three individual phenotypes), mixtures thereof (two phenotypes including double positives) or even a single dye at different concentrations (different strengths of a particular phenotype). In theory, the number of collection channels sequentially branching off from the waste channel is only limited by the desired throughput: the more channels used, the longer the pulse delay for the most downstream channels and hence the lower the overall throughput. However, for many biological screening applications, the number of different collection reservoirs is far more important than the throughput. For example, a setup as presented here could be used to sort individual droplets into the wells of a microwell plate for further downstream characterization (*e.g.* single-cell sequencing, biochemical or cell-based assays). Furthermore, sorting channels that sequentially branch off from the waste channel could also be replaced by a star-like geometry, which does not require long pulse delays and hence allows higher throughput. We believe that this would also allow the efficiency of the multi-way sorting device to be further increased, whose error rate is mostly limited by the exact timing of the sorting delay for each collection channel. This should not be the case for a symmetric, star-like geometry operating without sorting delays (similar to the ∼100% efficient 2-way sorting), or for which they could at least be equal and short for all collection channels. While electrode pairs running next to the channels are difficult to implement for such a geometry (because of space constraints), one could place electrode pairs above or below the sorting channels.^12^,^13^ This would even enable precise control of the trajectory of droplets along different paths within a single channel; however, much more complex manufacturing methods are required for such a device. In contrast to this, the setup shown here can be easily reproduced by standard soft lithography techniques and we hence look forward to see rapid spreading and further use of the technology.

## Materials and methods

### Software

A custom sorting software solution based on LabVIEW controlling a PXI-7854R FPGA card (National Instruments) was developed and can be downloaded from www.merten.embl.de/downloads.html. The software supports real time evaluation of the fluorescence signals and switching of up to eight uncoupled electrode pairs by a downstream multi-way switch (Fig. S3†). Analog signal filtering and baseline correction were performed by two long-pass filters per signal channel at the level of the FPGA card. The filters had cut-off frequencies of typically 100 Hz to 10 000 Hz and 0.005 Hz to 0.5 Hz, respectively.

### Electronic multi-way switch

Fast switching of multiple electrode pairs was achieved using an in-house high voltage switch (Fig. S3†). A Trek 623B amplifier was connected to the output OUT*n* (with *n* ranging from 1 to 8) and GND *via* the switch SW*n*. For sorting, output OUT*n* is disabled by switching off SW*n* and additionally switching on SW*n*-DISCH to rapidly release the remaining charge from the waste electrode. In parallel, OUT*n* (powering the respective sorting electrode) is switched on *via* SW*n*-MAIN for a pre-determined pulse duration before the charge is released *via* SW*n*-DISCH. Subsequently, the system returns to its default switch configuration (powering the waste electrode).

### 2-Way sorting

2-Way sorting was performed by generating 100 μm droplets containing 100 μM FITC directly on the sorting chip. The aqueous phase and QX 200 (Biorad droplet generation oil) were injected into the flow-focussing geometry at a rate of 20–200 μl h^−1^ and 900–1250 μl h^−1^, and the resulting droplets were spaced out by injecting Novec 7500 (3 M) at a rate of 400 μl h^−1^ further downstream. To evaluate the flow rate dependency of our setup at a droplet frequency of 13 Hz, we aspirated liquid from the waste outlet corresponding to 5, 6, 7, 11, 20, 30, 40, 50, 60, 70, 80, 85, 86, 87, 88 and 90% of the total flow rate (1320 μl h^−1^). Also, to take the effect of the droplet frequency into account, we repeated the experiments at ∼90 Hz and aspirated at a fraction of 5, 6, 7, 11, 20, 25, 30, 40, 50, 60, 70, 72, 75, 80, 85, 86, 87, 88 and 90% of the total flow rate. To mimic a conventional sorting setup, we further performed the experiment using only the electrode pair next to the collection channel and aspirated at a fraction of 50, 60, 65, 75, 78, 80, 85 and 90% of the total flow rate (note that for this approach, one always has to aspirate a higher volume fraction from the waste outlet). The applied input voltage (1.5 kV) for the electrodes was constant for all experiments, and the on-time of the sorting electrode was 10 ms for 13 Hz sorting and 5 ms for 90 Hz sorting. Every second droplet was test-sorted, to illustrate that manipulation on the level of a single droplet is possible, and because this setup stresses the switch maximally. Sorting efficiencies were also characterized in experiments using 1 : 2, 1 : 3 and 1 : 5 sorting modes at constant relative flow rates (aspirating 50% of the total flow from the waste outlet; Table 1).

### 4-Way droplet sorting

For movie analysis of the sorting process, droplets containing 187.5 μM cascade blue (CB; +2 g l^−1^ naphthol blue black, (NBB)), 250 μM FITC (+1.25 g l^−1^ NBB) and 187.5 μM FITC plus 125 μM CB (without light absorbing NBB, for which reason the fluorophore concentrations were decreased to get comparable signals) were generated. For analysis of the sorted fractions by fluorescence microscopy, droplets containing 187.5 μM CB, 20 μM Alexa 488 and 125 μM Alexa 594 were generated. For both approaches, droplets of ∼100 μm in diameter were generated by injecting the respective aqueous samples (400–500 μl h^−1^) and QX200 (Biorad droplet generation oil; 2000 μl h^−1^) into the drop makers shown in Fig. S1† (using only four of the eight available droplet makers). The resulting mixed emulsions were collected in a falcon tube (15 ml) and subsequently reinjected into the sorting device at a flow rate of 5 μl h^−1^ using a 250 μl Hamilton syringe. To achieve sufficient droplet spacing, QX200 was injected into the flow focussing module at a flow rate of 100 μl h^−1^. At the single inlet geometry further downstream, a mixture of 50% Novec 7500 and 50% QX200 was injected at a flow rate of 500 μl h^−1^ (sorting at 2 Hz) or 750 μl h^−1^ (sorting at 3 Hz). To achieve optimal performance, a syringe was connected to the waste outlet aspirating at a rate of 250 μl h^−1^ (2 Hz) or 400 μl h^−1^ (3 Hz). All flow rates were controlled using Harvard Apparatus PhD 2000 syringe pumps. Based on different distances from the detection point, the following delays were implemented before actuating the electrode pairs (sorting at 2 Hz/3 Hz): 260/150 ms (first electrode pair), 370/240 ms (second electrode pair) and 480/320 ms (third electrode pair). The input voltage was 1.25 kV and the pulse duration for switching electrode pairs was 20/40 ms.

## Supplementary Material

Supplementary information

## Figures and Tables

**Table 1:** Performance and efficiency of 2-way sorting (as determined by analyzing high-speed movies)

Sorting ratio	Droplets	Rate [Hz]	Efficiency [%]
1 : 2	761	153	100
1 : 3	802	161	100
1 : 5	778	157	100

**Table 2:** Flow rate independency of 2-way sorting at 80–100 Hz using one and two electrode pairs (as determined by analyzing high-speed movies; Fig. 1D)

Number of powered electrode pairs	Relative flow rates (waste/collection channel)	Efficiency^a^ [%]
One	50 : 50	0
	60 : 40	0
	65 : 35	0
	75 : 25	3.4
	78 : 22	41.5
	80 : 20	99.4
	85 : 15	100
	90 : 10	1.1
Two	11 : 89	0
	20 : 80	17.2
	25 : 75	53.3
	30 : 70	90.7
	40 : 60	100
	50 : 50	100
	60 : 40	100
	70 : 30	100
	72 : 28	85.5
	75 : 25	0.6
	80 : 20	0

a On average, more than 380 sorting events were analyzed for each data point shown in Fig. 1D.

**Table 3:** Sorting efficiency while flicking the tubing connected to the outlets (see also ESI Movies S12 and S13). On average, the sorting throughput was ~110 Hz and more than 600 sorting events were analyzed per experiment

Experiment	Efficiency when using one electrode pair [%]	Efficiency when using two electrode pairs [%]
#1	92.6	99.8
#2	85.7	100
#3	97.5	99.4
#4	92.9	98.4

**Table 4:** Performance and efficiency of 4-way sorting (as determined by analyzing high-speed movies)

Content	Channel	Delay [ms]	Rate [Hz]	Droplets	Efficiency [%]
FITC/CB	#1	260	2	36	97.2
FITC	#2	370		55	100.0
CB	#3	480		67	98.5
Sorting with inversed channel order					
CB	#1	260	2	50	100.0
FITC	#2	370		36	100.0
FITC/CB	#3	480		15	93.3

**Table 5:** Performance and efficiency of 4-way sorting (as determined by fluorescence microscopy of sorted fractions; Fig. 2F and G)

Content	Channel	Delay [ms]	Rate [Hz]	Droplets	Efficiency [%]
Sorting for different colours					
Alexa 594	#1	150	3	274	84.5
Alexa 488	#2	240		504	97.4
CB	#3	320		210	92.9
Sorting for different intensities					
12.5 μM Alexa 488	#1	150	3	333	94.6
25 μM Alexa 488	#2	240		205	92.7
50 μM Alexa 488	#3	320		154	93.5
