# Supplementary material for: Microfluidic train station: highly robust and multiplexable sorting of droplets on electric rails
Source: Lab Chip. Author manuscript; Available in PMC 2017 Sep 14. (PMC5544107; doi:10.1039/c6lc01544a)
Supplement: Supplementary information [file NIHMS73452-supplement-ESI.zip › ESI.docx]

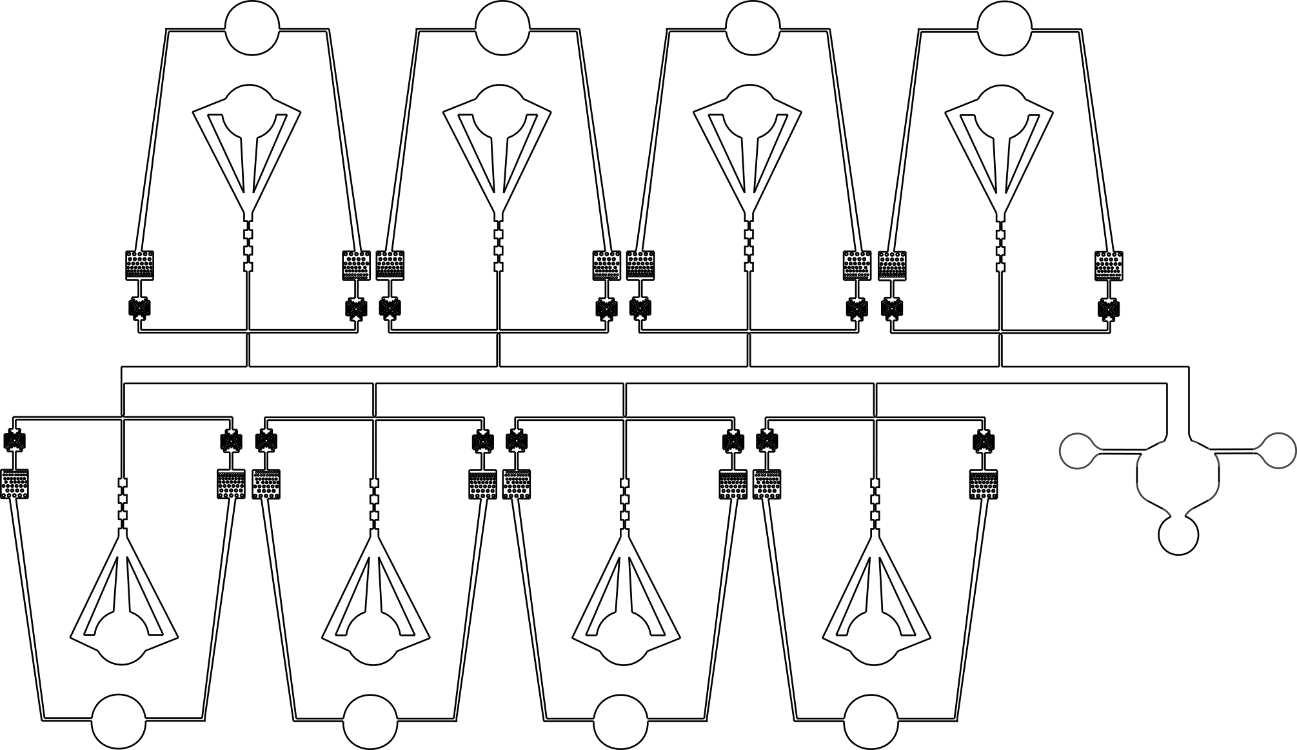


**Figure S1: Design of the droplet maker used in this work.** The design includes eight parallel droplet makers on one chip. This allows simultaneous creation, mixing and sample collection of mixed droplet emulsions.

**
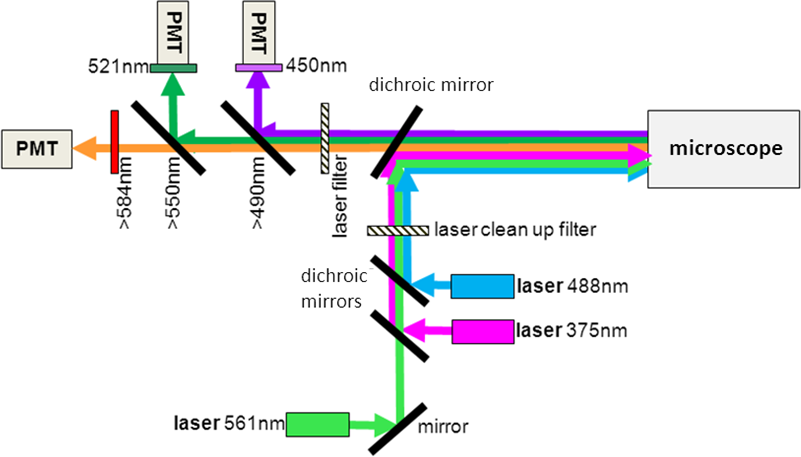
**

**Figure S2: Optical setup of the microfluidic work station used in this study.** 3 different lasers are used for excitation at 375 nm, 488 nm and 561 nm. Emission is measured at 450 nm, 521 nm and >584 nm.


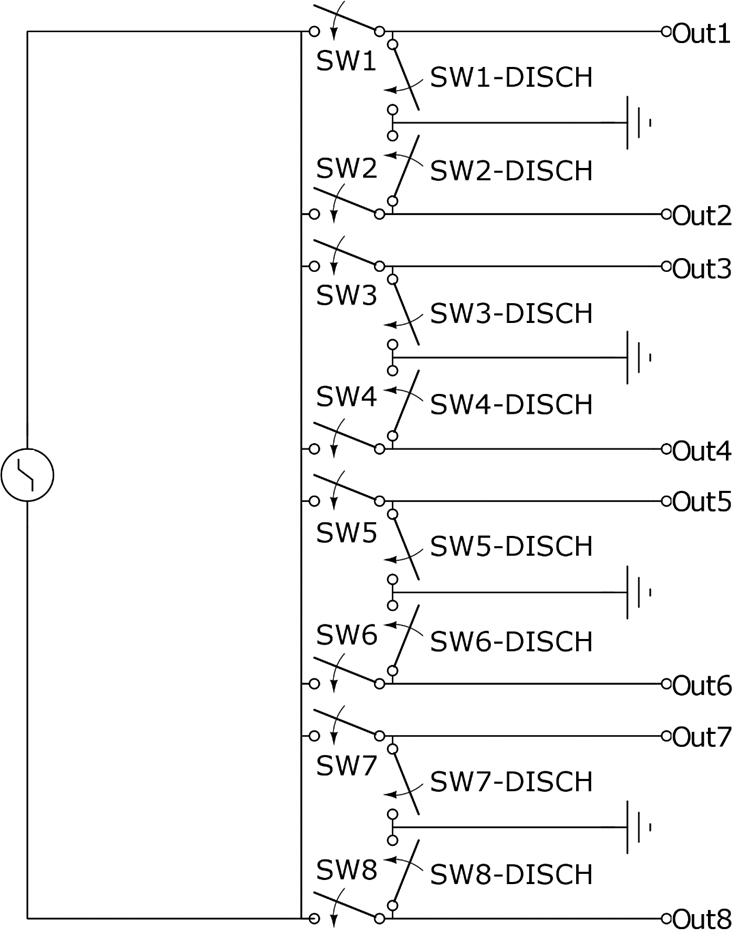


**Figure S3: Illustration of the multi-way switch**. Eight outputs (Out 1-8) can be connected to the electrodes of the microfluidic chip. A certain output “x” can be enabled by closing the corresponding switch “SWx” and opening the switch “SWx-DISCH”. If the electrode shall be disabled, the switch “SWx” is opened and the switch “SWx-DISCH” is closed, thereby rapidly discharging the electrode.

**
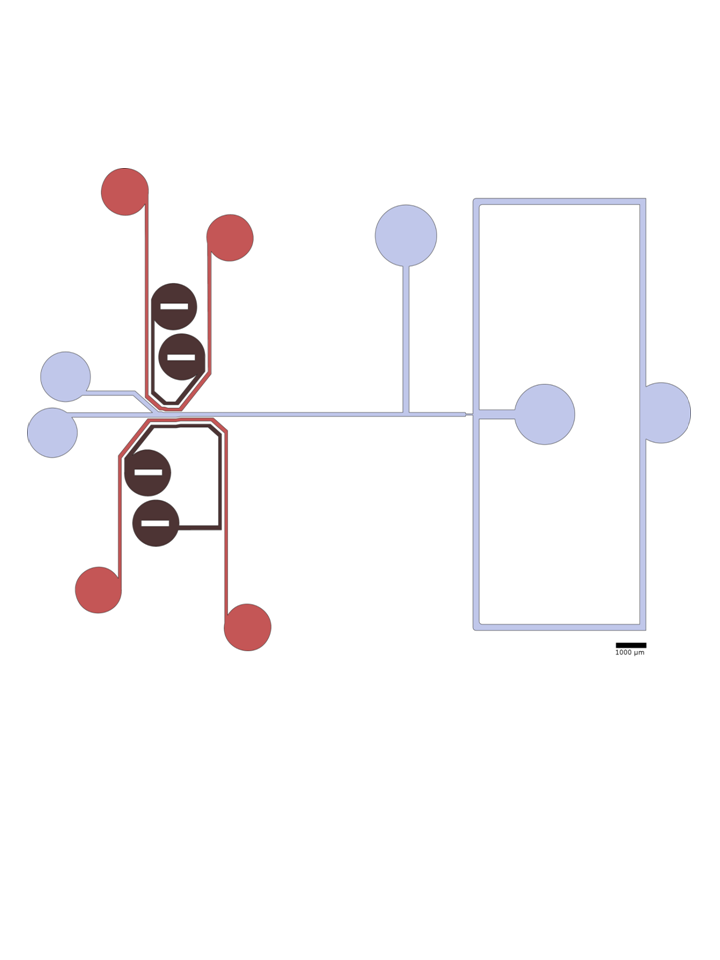
**

**Figure S4: Enlarged image of the 2-way sorting chip.** Flow channels are shown in light blue, power electrodes in red, ground electrodes in brown.

**
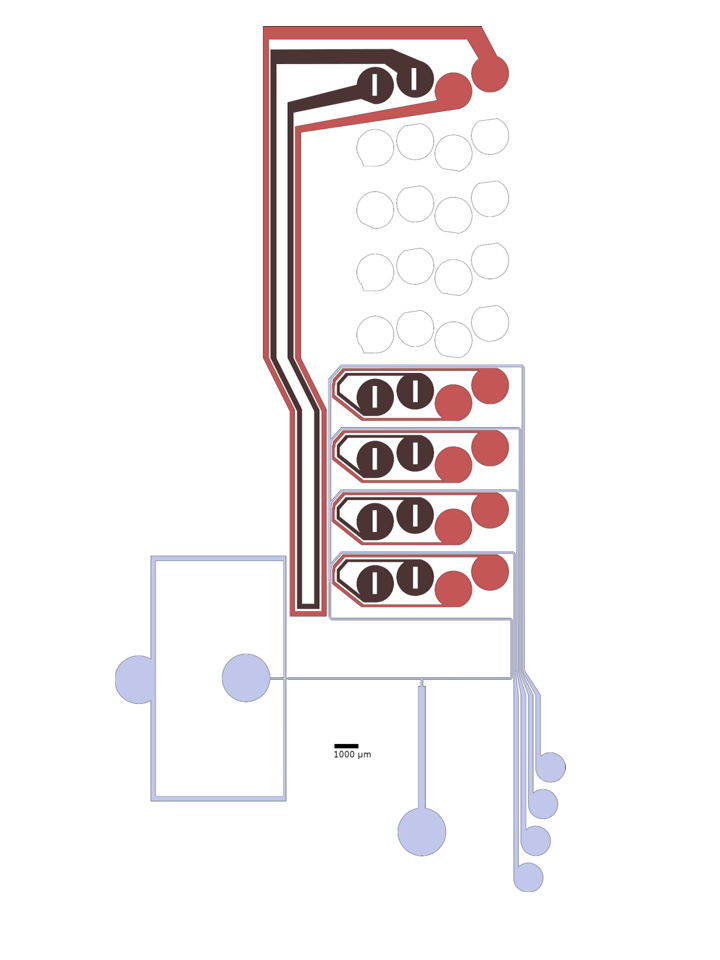
Figure S5: Enlarged image of the 4-way sorting chip.** Flow channels are shown in light blue, power electrodes in red, ground electrodes in brown.


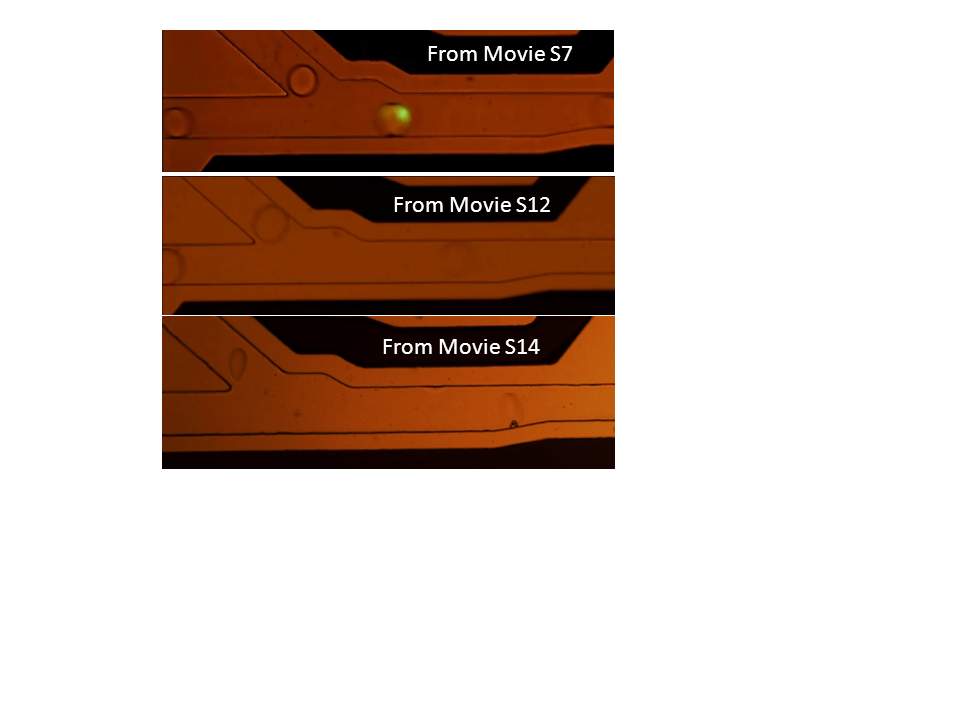


**Figure S6: Droplets of different sizes can be sorted without the need for changing the geometry of the chip.**

**Supplemental Table 1: Long-term sorting efficiency of 2-way sorting at constant relative flow rates and a throughput of ~65 Hz using two electrode pairs (as determined by analyzing high-speed movies).**

| **Time point of analysis** | **Droplets** | **Efficiency [%]** |
| --- | --- | --- |
| 0h | 485 | 100 |
| 1h | 493 | 100 |
| 2h | 586 | 100 |
| 2h | 448 | 100 |
| 3h | 604 | 100 |
| 3h | 552 | 100 |
| 4h | 454 | 100 |
| 4h | 486 | 100 |
| 5h | 551 | 100 |

**Supplemental Table 2. Effect of the continuously applied DEP force on droplet velocity (as determined by video analysis).**

| **Experimental Setup** | **Average droplet speed while waste electrodes are off [µm/ms]** | **Average droplet speed while waste electrodes are on [µm/ms]** | **Decrease in droplet speed**  **[%]** |
| --- | --- | --- | --- |
| Voltage = **1125V**  Total flow rate = 975 µl/h | 62.8 | 60.3 | 4.0 |
| Voltage **1688V**  Total flow rate = 975 µl/h | 61.6 | 55.8 | 9.4 |
| Voltage = **2250V**  Total flow rate = 975 µl/h | 62.0 | 53.7 | 13.4 |
|  | | | |
| Voltage = 1125V  Total flow rate = **975 µl/h** | 62.8 | 60.3 | 4.0 |
| Voltage = 1125V  Total flow rate = **1350 µl/h** | 78.1 | 76.5 | 2.0 |
| Voltage = 1125V  Total flow rate = **1725 µl/h** | 101.2 | 100.4 | 0.8 |
| Voltage = 1125V  Total flow rate = **2100 µl/h** | 123.9 | 118.3 | 4.5 |

**Movie S1: 242 Hz two electrode sort, aspirating with 50% of the total flow rate from the outlet.**

**Movie S2: 13 Hz two electrode sort, aspirating with 20% of the total flow rate from the outlet.**

**Movie S3: 13 Hz two electrode sort, aspirating with 50% of the total flow rate from the outlet.**

**Movie S4: 13 Hz two electrode sort, aspirating with 80% of the total flow rate from the outlet.**

**Movie S5: ~90 Hz two electrode sort, aspirating with 40% of the total flow rate from the outlet.**

**Movie S6: ~90 Hz two electrode sort, aspirating with 60% of the total flow rate from the outlet.**

**Movie S7: ~90 Hz two electrode sort, aspirating with 70% of the total flow rate from the outlet.**

**Movie S8: ~90 Hz one electrode sort, aspirating with 78% of the total flow rate from the outlet.**

**Movie S9: ~90 Hz one electrode sort, aspirating with 80% of the total flow rate from the outlet.**

**Movie S10: ~90 Hz one electrode sort, aspirating with 90% of the total flow rate from the outlet.**

**Movie S11: Effect of flicking the tubing connected to the outlet when using a single electrode pair for sorting.** The tubing was flicked six times while recording the movie.

**Movie S12: Effect of flicking the tubing connected to the outlet when using two electrode pairs for sorting.** The tubing was flicked six times while recording the movie.

**Movie S13: Effect of the continuous DEP force on droplet velocity.** 1688V were applied to the waste electrodes; a higher voltage than used in all sorting experiments (max 1500V).

**Movie S14: 1 in 3 sorting of small droplets.** Using aqueous and oil flow rates of 10 µl/h and 140 µl/h at the drop maker enabled the generation and sorting of particularly small droplets (~60µm in diameter).

**Movie S15 4-way sorting at 2 Hz.** Droplets contain 187.5 µM cascade blue (CB; + 2 g/l Naphtol Blue Black, dark grey), 250 µM FITC (+ 1.25 g/l NBB, light grey) and 187.5 µM FITC plus 125 µM CB (clear; without light absorbing NBB, for which reason the fluorophore concentrations were decreased to get comparable signals).
